# mRNA-to-protein translation in hypoxia

**DOI:** 10.1186/s12943-019-0968-4

**Published:** 2019-03-30

**Authors:** Nancy T. Chee, Ines Lohse, Shaun P. Brothers

**Affiliations:** Department of Psychiatry and Behavioral Sciences, University of Miami Miller School of Medicine, 1501 NW 10th Avenue, Miami, FL 33136 USA

**Keywords:** Hypoxia, mRNA-to-protein translation, HIF, Hypoxia-inducible factor, Cancer, HIF inhibitors

## Abstract

Cells respond to hypoxia by shifting cellular processes from general housekeeping functions to activating specialized hypoxia-response pathways. Oxygen plays an important role in generating ATP to maintain a productive rate of protein synthesis in normoxia. In hypoxia, the rate of the canonical protein synthesis pathway is significantly slowed and impaired due to limited ATP availability, necessitating an alternative mechanism to mediate protein synthesis and facilitate adaptation. Hypoxia adaptation is largely mediated by hypoxia-inducible factors (HIFs). While HIFs are well known for their transcriptional functions, they also play imperative roles in translation to mediate hypoxic protein synthesis. Such adaptations to hypoxia are often hyperactive in solid tumors, contributing to the expression of cancer hallmarks, including treatment resistance. The current literature on protein synthesis in hypoxia is reviewed here, inclusive of hypoxia-specific mRNA selection to translation termination. Current HIF targeting therapies are also discussed as are the opportunities involved with targeting hypoxia specific protein synthesis pathways.

## Introduction

Hypoxia is vaguely defined as the decrease in oxygen availability below normal tissue levels. Due to varying oxygen tensions in different tissues, what constitutes low oxygen conditions also varies [1, 2]. There are generally two types of hypoxia: acute and chronic. Acute hypoxia is a rapid and transient decrease in pO_2_ that may be caused by an obstruction of the airways, acute hemorrhaging or abrupt cardiorespiratory failure. If the stress is not alleviated, acute hypoxia can cause damage to those systems, contributing to the development of chronic hypoxia. Chronic hypoxia occurs when oxygen supply is limited for long periods of time. Chronic hypoxia is seen in solid tumors, where oxygen consumption outweighs oxygen influx [3]. Due to unstable homeostasis in solid tumors, cells can quickly cycle between normoxic and hypoxic states, adding another layer of microenvironmental complexity in cancer [4].

A core characteristic of the tumor microenvironment, hypoxia is present in all solid tumors and has been proposed to also influence liquid cancers [3, 5–8]. Although tumors are vascularized, rapid angiogenesis results in the formation of an ineffective and leaky vascular network often containing dead ends [9]. While the exterior cells of the tumor mass are more likely to receive sufficient oxygen, the core of the tumor lacks oxygen and generally displays areas of severe chronic hypoxia [10]. Hypoxic regions are not limited to the tumor core, and can occur throughout the mass even in close proximity to what histologically appears to be a functional blood vessel, suggesting that demand-to-supply imbalance contributes to hypoxic microenvironments [10].

Hypoxic tumor cells can survive due to changes in cellular processes partially mediated by the accumulation and activity of hypoxia-inducible factors (HIFs). Data support the hypothesis that chemotherapy and radiation resistance seen in cancers are at least partially due to increased HIF activity [11–14]. Hence, tumorigenesis may be inhibited by blocking HIF activity in these hypoxic cells, making HIFs an attractive target for treating some cancers [15–19]. HIFs are well-known as transcription factors. However, their role in mRNA-to-protein translation is also imperative to cell survival since the canonical protein synthesis pathway is impaired in hypoxia.

As one of the most energy-consuming processes in the cell, translation requires enormous amounts of ATP synthesized in healthy cells [20]. Cells metabolize glucose to generate ATP, a process that requires oxygen. Therefore, low oxygen supply results in decreased rate of global mRNA-to-protein translation in the cell due to decreased ATP availability. Because the canonical translation pathways require large amounts of ATP generated in the presence of oxygen, hypoxia limits this translation pathway, thus necessitating an alternative translation pathway to efficiently synthesize proteins in hypoxic environments [21, 22]. HIFs are major regulators of the alternative hypoxia-induced translation pathway activation.

### Hypoxia-inducible factors

HIFs are a family of proteins that mediate cellular adaptation to hypoxia. Heterodimeric HIF transcription factors consist of HIFα and HIFβ subunits. The HIFα subunits are cytosolic, constitutively synthesized and tightly regulated. The HIFβ subunit (aryl hydrocarbon receptor nuclear translocator (ARNT)), is a constitutively active DNA binding protein that remains in the nucleus.

The HIFα family of proteins is comprised of three subtypes: HIF1α, HIF2α and HIF3α. HIF1α is ubiquitously expressed at low, basal levels in all tissues in healthy individuals in normoxia. HIF1α expression increases with transient, acute hypoxia exposure in most tissues and decreases to basal levels after reaching its maximum expression [23, 24]. HIF2α and HIF3α expressions are more tissue specific. HIF2α is preferentially expressed in organs that experience greater hypoxia, such as the pancreas, liver and kidneys [25, 26]. HIF2α increases expression with prolonged, chronic hypoxia exposure, suggesting that HIF1α and HIF2α subtypes play different roles in cellular adaptation to acute and chronic hypoxia [23, 24]. HIF3α is preferentially expressed in the heart, lungs, cerebellum and eyes and has been found to inhibit HIF1α and HIF2α activity [27]. The role of HIF3α in hypoxic physiology remains to be elucidated. HIFα expression increases with continuous exposure to hypoxia and the duration of exposure to reach maximal HIF expression depends on the tissue type [23].

Structurally, HIF1α and HIF2α are highly homologous, containing the same motifs and domains. They both contain basic-helix-loop-helix (bHLH) and Per-Arnt-Sim (PAS) domains, which are required for DNA-binding and heterodimerization with ARNT in response to hypoxia, respectively [28]. HIF1α and HIF2α also contain transcriptional activation domains at the N-terminus (N-TAD) and the C-terminus (C-TAD) that are required to activate transcription of hypoxia-inducible genes and are subject to regulation by hydroxylation in normoxia [17]. The most differences in structure of the two isoforms are within the N-TAD region. The N-TAD is responsible for recognizing transcriptional target genes and due to the differences found in the N-TAD between HIF1α and HIF2α, these proteins may target sets of different genes [29]. These two subtypes also contain an oxygen-dependent degradation domain (ODDD), required for regulation by oxygen-dependent proteins that degrade the HIFs in normoxia [30–32].

**Fig. 1:**
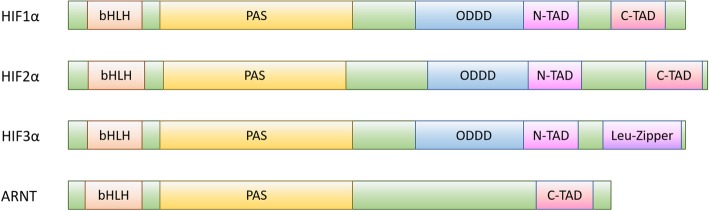
Hypoxia-Inducible Factors structural schematic. bHLH: basic helix-loop-helix; PAS: Per-Arnt-Sim (period circadian protein, aryl hydrocarbon receptor nuclear translocator protein, single-minded protein) domain; ODDD: oxygen-dependent degradation domain; N-TAD: N-terminus transcriptional activation domain; C-TAD: C-terminus transcriptional activation domain; Leu-Zipper: leucine-zipper domain

HIF3α is structurally similar to HIF1α and HIF2α as it contains bHLH-PAS domains, ODD domains and N-TAD, as seen in Fig. 1. Unlike HIF1α and HIF2α, however, HIF3α lacks the C-TAD, which plays a role in HIF stabilization and transcription activation in HIF1α and HIF2α. The absence of C-TAD in HIF3α suggests a secondary function independent of its transcriptional activity [17, 33]. Also indicative of a secondary function, HIF3α contains a unique leucine zipper domain, which may facilitate DNA binding and protein-protein interactions. HIF3α is subject to extensive alternative splicing that yields at least six different splice variants that may target different genes or have functions that are entirely independent from transcription [34]. Some of these splice variants, especially HIF3α4, negatively regulate the transcriptional roles of HIF1α and HIF2α by direct binding [35]. Different splice variants of HIF1α that lack the ODDD and TAD have also been found, although the functions of these variants have yet to be elucidated [36].

Like the HIFα subunits, the ARNT subunit contains bHLH and PAS domains. However, it does not contain the N-TAD region or the ODD domain, suggesting its oxygen-independent expression. ARNT is a nuclear translocator involved in many different cellular processes that aid protein translocation from the cytosol or the nuclear membrane into the nucleus. Hence, ARNT is ubiquitously and constitutively expressed. It is involved in cancer progression, chemotherapy resistance, wound healing and immune response pathophysiology [37–40].

HIFs are mostly known for their function as transcription factors, altering the transcriptome to mediate cellular response to hypoxia. Generally, HIF transcriptional target genes stimulate cell survival, metabolism, angiogenesis, metastasis and pH regulation in adaptation to low oxygen and increased intracellular acidity. Target genes include *EPO, VEGF, GLUT-1, OCT4, CXCR4* and *LDH*, among a plethora of others [41, 42]. Despite the structural and sequence homology between HIF1α and HIF2α, these two proteins target distinct genes for transcription, as well as some overlapping genes. The role of HIF3α in transcription is not as well elucidated as its counterparts. HIF3α appears to be a negative regulator of gene expression in hypoxia by preventing HIF1α mediated transcription activation [35, 43]. HIF3α reduces HIF1 and HIF2α activity by competing for HIF1β subunit binding [43]. HIF3α also activates transcription of genes that are not targeted by HIF1α or HIF2α, such as *LC3C, REDD1 and SQRDL* [44].

#### HIF regulation

HIF1α and HIF2α are well characterized in their roles as transcription factors [41]. In hypoxia, HIFα subunits accumulate and translocate to the nucleus where it dimerizes with ARNT. The HIF/ARNT heterodimer recruits p300/CBP, forming a complex that binds to the hypoxia response elements (HRE) in promoter regions to activate target gene transcription [17, 41]. To prevent increased HIF activity in normoxia, HIFs are tightly regulated by different pathways and enzymes. HIFs undergo proline hydroxylation, ubiquitination, SUMOylation, S-nitrosylation, asparagine hydroxylation and phosphorylation to promote HIF degradation.

One of the major HIF regulatory proteins is HIF-prolyl hydroxylase 2 (HIF-PH2) that belongs to the prolyl hydroxylase domain enzyme (PHD) family. PHDs are a major oxygen-sensing protein family that, upon binding to oxygen, hydroxylates different target protein to initiate a cellular response. HIF-PHD hydroxylates HIFs at proline residues (pro^402^ and pro^564^ in HIF1α, pro^405^ and pro^531^ in HIF2α, pro^492^ in HIF3α) in the HIF ODDD [45–48]. These modifications facilitate the recruitment of von Hippel-Lindau ubiquitin ligase complex (pVHL-E3 ligase complex) that ubiquitinates HIFα, promoting proteasomal degradation [46].

HIF1α is also subject to SUMOylation, which ultimately stabilizes the protein and enhances its transcriptional activity. HIF1α is SUMOylated at residues lys^398^ and lys^477^ in the ODD domain and may modulate other post-translational modifications, such as ubiquitination, to increase stability and activity in vitro and in vivo [49, 50]. A SUMO moiety is transferred from the E1-activating enzyme to the E2-conjugation enzyme, particularly Ubc9, which then carries the SUMO moiety to the target protein [51]. SUMO E3-ligase enzymes then mediate the final transfer of the SUMO from the E2-conjugation enzymes to the HIF1α lysine residues. While the SUMOylation of HIF1α increases its transcriptional activity, HIF1β is also SUMOylated at lys^245^ which decreases HIF1α transcriptional activity [52]. While it is generally accepted that SUMOylation in hypoxia leads to HIF1α stabilization and increased transcriptional activity, there are studies that demonstrate increased HIF1α degradation after SUMOylation, making the underlying biology unclear [53]. SUMOylation also has an important role in promoting HIF2α transcriptional activity. Hypoxia associated factor (HAF), a HIF1α-E3 ligase, is SUMOylated under hypoxic conditions and binds to the DNA upstream of the HRE in the promoter region of HIF2α target genes. This binding promotes HIF2α binding to the HRE, activating its transcriptional activity [54].

As hypoxic exposure progresses, nitric oxide (NO) levels also increase, leading to HIFα S-nitrosylation. HIF1α is S-nitrosylated at cysteine residues cys^520^ and cys^800^. S-nitrosylation at cys^520^, which lies within the ODD domain of HIF1α, increases the stability of the protein and impairs degradation by blocking prolyl hydroxylation and preventing ubiquitination. S-nitrosylation of residue cys^800^ promotes HIF1α binding to transcriptional co-factors, such as p300 and CBP, ultimately enhancing its transcriptional activity [55–57].

**Fig. 2:**
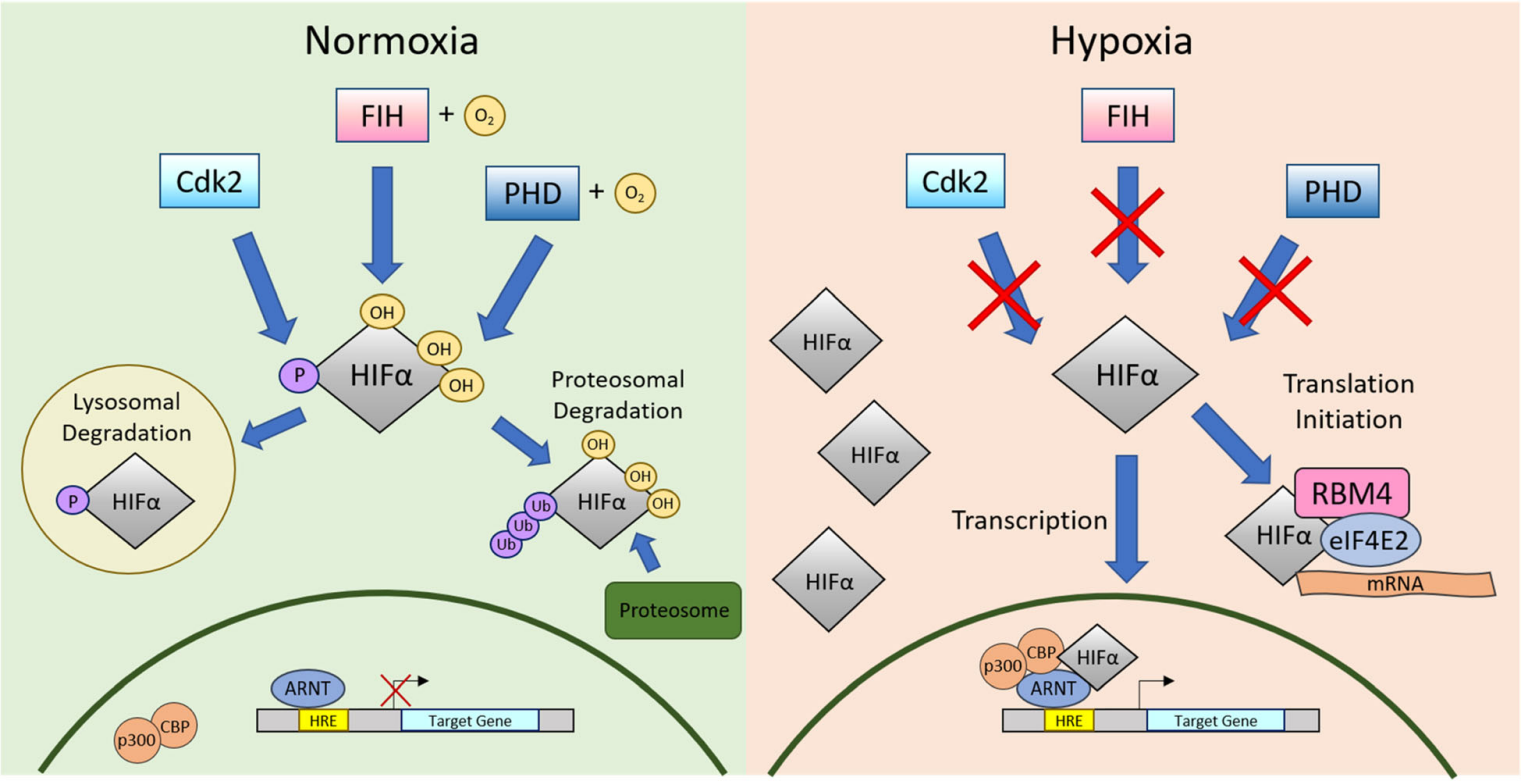
HIF regulation in normoxia and hypoxia. HIFα: hypoxia-inducible factor alpha; PHD: prolyl hydroxylase domain enzyme; FIH: factor inhibiting HIF; Cdk2: Cyclin dependent kinase 2; O2: oxygen molecule; ARNT: aryl hydrocarbon receptor nuclear translocator; HRE: hypoxia response element; p300: protein 300; CBP: CREB-binding protein; RBM4: RNA-binding motif protein 4; eIF4E2: eukaryotic initiation factor 4E2; OH: hydroxyl group; P: phosphate group; mRNA: messenger RNA; Ub: ubiquitin

Additionally, HIFα transcriptional activity is inhibited in normoxia by an asparagine hydroxylase, factor-inhibiting hypoxia-inducible factor (FIH). FIH catalyzes HIFα (asp^803^) hydroxylation in the C-TAD, the binding sites of co-transactivators p300/CBP that promote transcription of HIF target genes [58]. Hydroxylation of C-TAD prevents p300/CBP co-activators from binding to HIFs, ultimately blocking hypoxia-response element promoter binding [59, 60]. Because HIF-PHD and FIH use oxygen as co-substrates to hydroxylate HIFs, hydroxylation cannot occur in hypoxia, causing HIF stabilization and accumulation. HIFs can translocate to the nucleus to initiate transcription or can remain in the cytoplasm to initiate translation of hypoxia-responsive proteins (Fig. 2) [3, 61]. Ineffective or faltered HIF regulation by PHDs or FIH may lead to cancer [62–65].

HIF1α is also regulated by cyclin-dependent kinase 2 (Cdk2) cell-cycle regulator protein. Cdk2 phosphorylates ser^668^ of HIF1α in normoxia, inhibiting proteasomal degradation and activating lysosomal degradation [59]. Initiating lysosomal degradation as opposed to proteasomal degradation ensures a secondary mechanism of HIF regulation in normoxia. In hypoxia, Cdk2 is inhibited, allowing HIF1α to accumulate to initiate cellular responses. Another cell cycle regulator protein Cdk1 also phosphorylates HIF1α ser^668^ to promote lysosomal degradation in normoxia. In hypoxia, accumulated HIF1α bind to and sequester Cdk1, inhibiting the lysosomal degradation pathway [59, 66].

In addition to these methods of HIFα regulation by other proteins, non-coding RNAs also play an important role in mediating cellular response to hypoxia. One of the most well-elucidated non-coding RNAs in hypoxia are micro-RNA-429 (miRNA-429) and micro-RNA-210 (miRNA-210), which has been shown to create a negative feedback look with HIF1α [67, 68]. These two miRNAs have been shown to directly bind to the 3′ UTR of the HIF1α gene, ultimately decreasing the expression of HIF1α. Interestingly, these miRNAs are also the target genes of HIF1α, creating a negative feedback look of HIF1α expression in hypoxia. HIFs are also regulated by hypoxia-responsive long non-coding RNA (HRL) [69, 70]. HRLs have a variety of functions in hypoxic cancers as they have been associated with increased tumorigenesis, ionizing radiation therapy resistance and metastasis [69–71]. HRLs are transcriptional targets of HIFs and unlike miRNAs, HRLs create a positive feedback by stabilizing HIFs by disrupting the HIF-VHL interaction, thus resulting in HIF accumulation [72].

### mRNA-to-protein translation and hypoxia

Hypoxia significantly alters general cellular processes that maintain housekeeping functions. While transcription and transcriptomic changes in hypoxia are relatively well elucidated, that of translation is less well-known as much of it remained a mystery until 2012. Uniacke et al. discovered the mechanism of protein synthesis in hypoxia that is directly mediated by HIF2α. This discovery opened doors to further understanding the mechanisms and regulations of translation in hypoxia.

mRNA-to-protein translation consists of three steps driven by eukaryotic translation factors: initiation by initiation factors (eIFs), elongation by elongation factors (eEFs) and termination by release factors (eRFs). Translation factors that promote each step are generally active in normoxia though some are inactive in hypoxia. Cells adapt to these hypoxia-induced changes by activating alternative transcription pathways and protein synthesis machinery to continue to synthesize proteins necessary to promote cell survival in low energy and low oxygen environments.

#### Hypoxia specific mRNA translation

Hypoxic protein synthesis is geared towards adaptation that is initiated through mechanisms of mRNA selection for translation. There are several proposed mechanisms that contribute to mRNA selectivity in hypoxia: upstream open-reading frame (uORF)-mediated mRNA regulation, endoplasmic reticulum-mediated mRNA selection, IRES-dependent translation initiation and the presence of ribosomal hypoxia-response elements (rHRE) in the mRNA recognized by the hypoxic translation machinery [73–77].

uORFs are short sequences that lie within the 5′ UTR region upstream of the protein coding sequence start codon, also called the main open-reading frame (mORF). The uORF is an essential cis-acting translation regulatory component that interacts with proteins that promote mORF translation or interacts directly with the ribosome, ultimately preventing mORF translation [78, 79]. Some 40–50% of all human mRNA transcripts contain at least one uORF that regulates mORF translation [78]. uORF regulation can decrease protein expression by 30 to 80% of its expression in normoxia [80]. In hypoxia, uORFs regulate HIF-mediated gene expression changes by allowing the scanning ribosome to bypass the uORF start codon, uAUG, allowing for mAUG recognition and mORF translation [81]. Translation of some mRNAs, such as *EPO, GADD34* and *VEGF*, rely on the presence of uORFs to activate translation distinctly in hypoxia and not as significantly in normoxia [77, 81–83].

Another mechanism that results in selective mRNA translation in hypoxia is the partitioning and recruitment of mRNAs to the endoplasmic reticulum (ER) [77]. Many mRNAs transcribed in hypoxia contain highly conserved 5′ and 3′ UTR elements that promote mRNA localization to the ER, where translation takes place [73, 74, 77]. Signal recognition particles (SRPs) recognize and bind to sequences in the conserved untranslated region (UTR) of mRNA to deliver it to the SRP-binding proteins present in the ER membrane [84]. Genes that localize to the ER in hypoxia for translation include *VEGF*, *HIF1* and *P4HA1* [77]. The localization of specific mRNA, including HIF target genes, to the ER in response to hypoxia further contributes to hypoxia-specific proteomic adaptations.

Selective hypoxia-responsive mRNA translation also occurs by the direct binding of the ribosome to internal ribosome entry sites (IRES). IRES are short sequences at the mRNA 5’UTR that promote ribosome recruitment without cap-binding translation initiation machinery [85, 86]. IRES vary in sequence among different genes and are also proposed to fold into secondary structures that promote ribosomal recruitment and binding [87]. IRES are mainly found in viral mRNA though some eukaryotic genes also harbor this sequence for selective translation initiation in response to stress, including hypoxia. Some genes known to utilize IRES-dependent translation in hypoxia include *VEGF* [88], human fibroblast growth factors (*FGF*) [89], insulin-like growth factors (*IGFs*) [90], *eIF4G* [91], platelet-derived growth factors (*PDGF*) [92] and proto-oncogene *C-MYC* [87, 93, 94]. While IRES-mediated protein synthesis is active and may partially explain the specificity of mRNA translated in hypoxia, IRES-mediated protein synthesis accounts for less than 1% of the level of cap-binding dependent mRNA-to-protein translation in hypoxia, a prevalence that is likely too low for cell survival [94]. Hence, IRES-mediated mRNA-to-protein translation is not sufficient to account for all translated proteins in hypoxia and an alternate pathway must exist.

While these mechanisms of mRNA selection for translation do not change in hypoxia compared to normoxia, genes containing uORFs or IRES regions in the mRNA rely on hypoxia for translation initiation. They are crucial to contributing to proteomic changes that mediate cellular response to hypoxia by selecting mRNA for translation initiation.

#### Translation initiation

In normoxia, mRNA-to-protein translation initiation is a concerted process involving mRNA activation by eukaryotic initiation factors (eIFs) and pre-initiation complex (PIC) recruitment. PIC consists of the 40S small ribosome subunit and an initiation tRNA charged with methionine (met-tRNA_i_) that recognizes the AUG start codon in the mRNA. PIC formation is catalyzed by eIF1, eIF1A, eIF2, eIF3 and eIF5. eIF1 and eIF1A are responsible for inducing an “open” conformational change to the 40S ribosome subunit to prevent the met-tRNA_i_ from binding to the A-site and promote its binding to the P-site [95]. eIF2 is a GTPase that forms a ternary complex with the met-tRNA_i_ and GTP [96]. eIF2 consists of three subunits, eIF2α, eIF2β and eIF2γ [96]. eIF2α contains a regulatory region in which ser^51^ phosphorylation regulates function. eIF2γ binds to GTP and hydrolyses the nucleotide to GDP. eIF2β mediates the exchange of GDP for a new GTP, promoting ternary complex formation and interacts with other initiation factors and the mRNA. eIF2 is active when eIF2α is not phosphorylated at ser^51^, as is the case in normoxia. In hypoxia, eIF2α is phosphorylated by kinases such as protein kinase R (PKR)-like endoplasmic reticulum kinase (PERK) [96].

PERK is an endoplasmic reticular kinase that “monitors” cell homeostasis by sensing ER stress and stress-induced protein unfolding in the ER, initiating the unfolded protein response (UPR) in cells. When activated, PERK ultimately inhibits global mRNA-to-protein translation [97]. While inactive in normoxia, PERK is hyperphosphorylated in hypoxia, which phosphorylates eIF2α. Phosphorylated eIF2α inhibits eIF2 GTPase function and prevents the ternary complex formation and recruitment of met-tRNA_i_ to the 40S ribosome and 43S PIC formation [96, 97]. PERK activation in the UPR pathway promotes preferential translation of mRNA that encode stress-responsive factors to restore cellular homeostasis [98]. This ultimately inhibits mRNA cap-binding in mRNA-to-protein translation initiation, promoting energy conservation and redirection of the energy conserved in cells to increase expression of cell survival genes. Interestingly, a rapid increase of eIF2α phosphorylation occurs in acute hypoxia but is reversed in prolonged hypoxia exposures [99]. eIF2α may slowly be de-phosphorylated and may become active in chronic hypoxia to mediate long-term adaptation and survival in hypoxia.

In parallel to PIC formation in normoxia, the mRNA translation is activated by eIF4E binding. eIF4E is a protein in the eIF4F complex that recognizes and binds to the 7-methyl-guanine cap structure at the 5′ end of the mRNA [100]. The eIF4F complex also consists of eIF4A and eIF4G proteins which remove mRNA secondary structures to allow for more conducive PIC binding to the 5′ end of the mRNA. eIF4G also binds to a poly-(A) binding protein (PABP), which associates with the 3′ poly-adenylated mRNA tail end. This was initially thought to cause the mRNA to fold into a loop structure [101–103]. However, recent research show that few mRNAs actually form this “closed-loop structure”; rather, mRNA bound to the eIF4F complex and not PABP form the loop structure, while mRNA bound to PABP, which consist of most mRNAs, do not [104, 105]. Further elucidation regarding the use of the “closed-loop structure” of mRNA in translation will be necessary. The eIF4F complex recruits the pre-assembled PIC to the 5′ end of the mRNA, forming the 48S ribosome-mRNA complex [106]. PIC scans the mRNA from the 5′ end to the 3′ end until the met-tRNA_i_ identifies and binds to the AUG start codon. Met-tRNA_i_ binding to the start codon causes eIF2 hydroxylation, which releases eIF proteins from the 48S complex and promotes the binding of the 60S large ribosome subunit to initiate translation elongation [107].

Cap-dependent translation initiation is regulated by mammalian target of rapamycin (mTOR) [108, 109]. mTOR is a protein kinase that phosphorylates target protein serine/threonine residues to ultimately promote cellular growth, proliferation and survival [109]. One mTOR complex 1 (mTORC1) target protein is the mRNA-to-protein translation repressor 4E binding protein (4E-BP), which sequesters eIF4E upon activation. 4E-BP phosphorylation by mTORC1 in normoxia allows eIF4E to bind to other initiation factors to begin protein synthesis [108]. While the mTORC1 pathway may be overactive in cancers, leading to dysregulated cell cycles and proliferation, hypoxia inhibits mTOR activity via REDD1 and AMPK activation [110, 111]. mTORC1 inhibition in hypoxia leads to the de-phosphorylation and activation of 4E-BP to continually sequester eIF4E [108, 109]. Hence, mTORC1 inactivation in hypoxia inhibits eIF4E at the translation initiation step. This has the effect of decreasing global mRNA-to-protein translation rate.

However, cells must continue to generate proteins that promote survival and adaptation under hypoxic stress. With the inhibition of mTORC1-mediated canonical translation mechanisms, cells activate alternative translation pathways that first begin with selective mRNA recruitment and translation initiation.

**Fig. 3:**
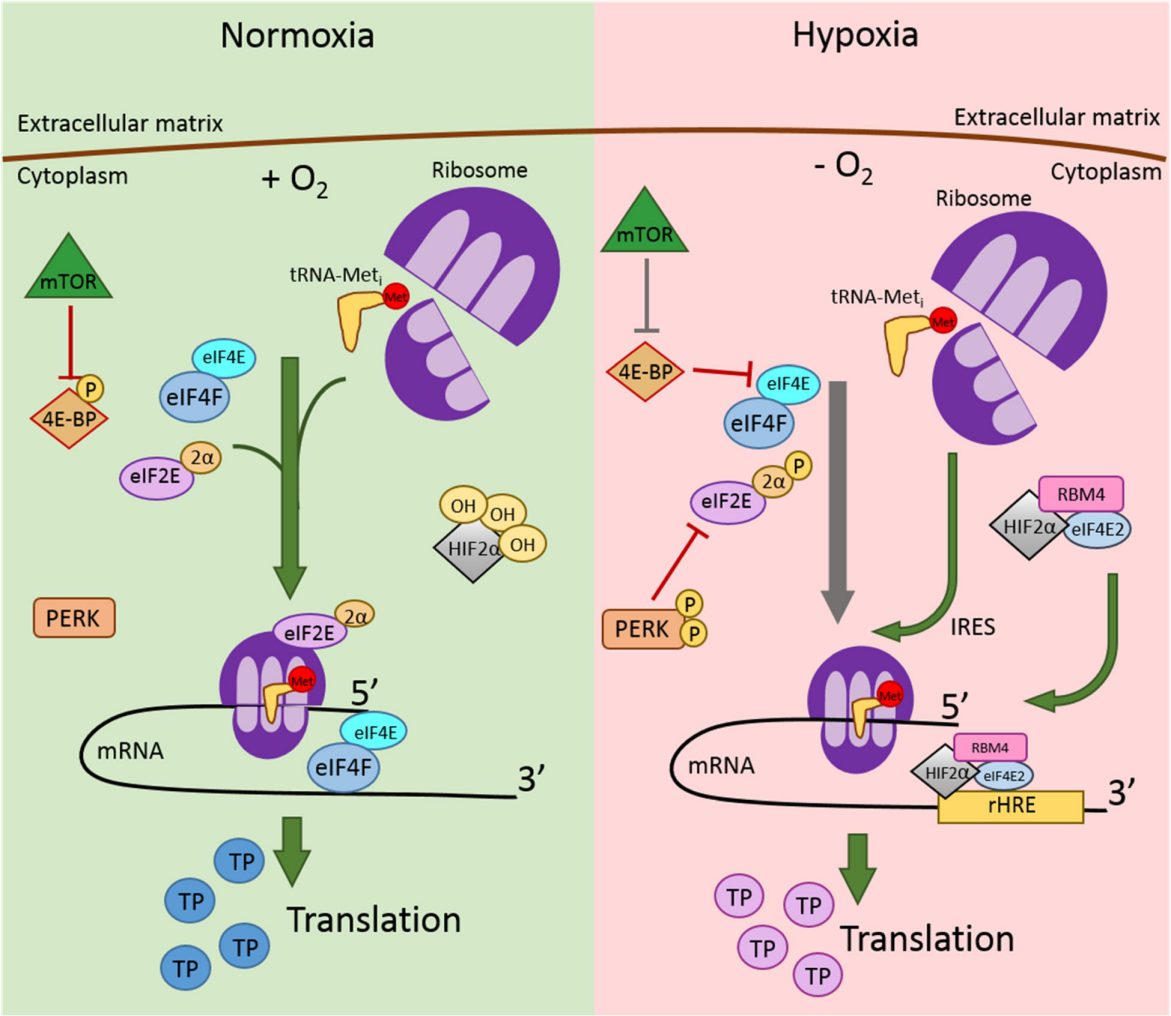
mRNA-to-protein translation initiation in normoxia and hypoxia. tRNA-Meti: transfer ribonucleic acid charged with initiation methionine; Met: methionine; eIF4E: eukaryotic initiation factor 4E; eIF4F: eukaryotic initiation factor complex 4F; eIF2E: eukaryotic initiation factor 2E; 2α: eukaryotic initiation factor 2 subunit α; mTOR: mammalian target of rapamycin; O2: oxygen; 4E-BP: eukaryotic initiation factor 4E binding protein; P: phosphate; PERK: protein kinase R (PKR)-like endoplasmic reticulum kinase; HIF2α: hypoxia-inducible factor 2α; OH: hydroxyl group; mRNA: messenger ribonucleic acid; TP: target protein; RBM4: RNA binding motif protein 4; eIF4E2: eukaryotic initiation factor 4E2; rHRE: RNA hypoxia response element

To provide insight into this seeming paradox of active mRNA-to-protein synthesis in hypoxia, Uniacke et al. discovered that HIF2α not only functions as a transcription factor in hypoxia, but also functions as a cap-dependent translation initiation factor in the absence of oxygen (Fig. 3) [22]. Hypoxia promotes the formation of a translation initiation complex that includes HIF2α, RNA-binding protein RBM4 and eIF4E2 [22]. The complex is assembled at the 3’UTR of the mRNA by recognition of a hypoxia response element (rHRE), identified as the sequence CG(G). RBM4 is first recruited to the rHRE, followed by HIF2α and eIF4E2, a homolog of eIF4E. The RBM4/HIF2α/eIF4E2 complex on the 3’UTR then interacts with the mRNA 5’cap [22].The complex binds to other initiation factors, namely eIF4A and eIF4G3, forming the eIF4F^H^ complex, which recruits ribosomes for translation [22, 112]. Cells appear to form the eIF4F^H^ complex only for hypoxic translation initiation; when RBM4, HIF2α or eIF4E2 are knocked down, the hypoxic cells are less viable. However, when one of those factors are inhibited in normoxic cells, no changes in global protein synthesis were observed [22, 112]. The discovery that hypoxic cells utilize a separate cap-dependent, oxygen-independent translation initiation mechanism has implications for hypoxic-specific cancer therapies.

Three different classes of mRNA appear in the hypoxia framework: class I consists of genes that are downregulated in hypoxia compared to normoxia; class II genes are oxygen-independently expressed genes and are not affected by hypoxia; and class III consists of genes that are upregulated in hypoxia [75]. Class III genes may be preferentially expressed in hypoxia due to the presence of the rHRE region in the mRNA that recruits the eIF4F^H^ complex to initiate cap-dependent translation [75]. Because only select gene mRNA transcripts contain the rHRE element, its role in recruiting the eIF4F^H^ complex to initiate translation significantly contributes to the specificity of protein expression in hypoxia. eIF4F^H^ complex may mediate a major pathway for hypoxic protein synthesis pathway analogous to the normoxic eIF4F complex.

#### Translation elongation

In normoxia, protein elongation is mediated by eukaryotic elongation factors (EFs). To begin the elongation step of mRNA-to-protein translation, eEF1A, a GTPase, binds to a GTP and an amino acid-bound tRNA (charged tRNA). This complex moves into the “A” site of the ribosome while the “P” site is occupied by the met-tRNA_i_ from the translation initiation step. If the charged tRNA anticodon in the “A” site matches the codon on the mRNA, eEF1A hydrolyzes the GTP and departs the complex, allowing the peptide bond to form between the charged tRNA in the “P” site and the incoming amino acid-tRNA in the “A” site. Upon peptide bond formation, the tRNA in the “A” site with the growing peptide bond will move to the “P” site. This movement is mediated by another elongation factor eEF2, a GTPase that translocate the tRNA from the “A” site to the subsequent position in the ribosome upon GTP hydrolysis. When the tRNA is in the correct “P” site, eEF2 releases from the “A” site of the ribosome, leaving it vacant for the next tRNA to match the following codon on the mRNA. In this process, eEF2 appears to be the only protein differentially regulated in hypoxia.

The rate of mRNA-to-protein translation elongation is regulated by eEF2 kinase (eEF2K). eEF2K is a unique calcium/calmodulin-binding kinase that regulates eEF2. eEF2K, when activated, phosphorylates and inhibits eEF2 activity thus inhibiting protein elongation when the cell is under stress [113]. The decreased rate of translation elongation may be imperative for cell survival since it allows cells to conserve energy and redirect the limited energy. In hypoxia, eEF2K is activated and phosphorylates eEF2, decreasing protein elongation rates [113, 114].

eEF2K activity is regulated by the binding of calcium/calmodulin complex and by proline hydroxylation. Under cell stress, the interaction between eEF2K and calcium/calmodulin facilitates eEF2K(thr^348^) autophosphorylation. The activated eEF2K then phosphorylates eEF2(thr^56^), inactivating the elongation factor and inhibiting translation elongation. It was initially believed that mTORC1 was the sole regulator of eEF2K in hypoxia that resulted in translation elongation downregulation [114]. However, eEF2K is also regulated by prolyl hydroxylation by prolyl hydroxylase 2 (PH2), a member of the PHD enzyme family [113]. In normoxia, PH2 uses oxygen as a co-substrate to hydroxylate eEF2K(pro^98^), preventing protein activation. This allows for eEF2 to remain unphosphorylated allowing translation elongation. In hypoxia, however, PH2 activity in eEF2K regulation is impaired due to the lack of oxygen co-substrate, allowing eEF2K to bind to calcium/calmodulin and leading to eEF2 phosphorylation and inactivation, decreasing the rate of global protein synthesis [113, 115–119].

It is well established that global mRNA-to-protein translation elongation rates are significantly decreased due to eEF2 inhibition by eEF2K activation in hypoxia [22]. However, the mechanism in which translation elongation occurs in hypoxia despite eEF2 inhibition remains unknown. It will be interesting to find out how hypoxic cells accomplish translation elongation when the mechanism is eventually discovered.

#### Translation termination

mRNA-to-protein translation is terminated by release factors 1 and 3 (eRF1 and eRF3). eRF1 is a structural homolog of tRNAs, consisting of a codon binding site that recognizes the three stop codons: UAA, UGA and UAG. At the end of translation elongation, the ribosome shifts down the mRNA to the stop codon. An incoming eRF1 enters the A site and binds to the stop codon, promoting the recruitment of eRF3, a GTPase that binds to eRF1. eRF3 then hydrolyzes the end of the polypeptide chain protruding from the P site. This hydrolysis releases the newly synthesized protein from the ribosome and allows dissociation of the ribosome and mRNA complex [120].

The rate of translation termination is controlled by posttranslational modifications of eRF1. eRF1 contains a highly conserved Asparagine-Isoleucine-Lysine-Serine (NIKS) sequence at the N-terminus that is hydroxylated by an oxygenase Jumonji domain-containing 4 (Jmjd4) [121, 122]. eRF1 hydroxylation is required for optimal translation termination rates in normoxia. In hypoxia, eRF1 hydroxylation is decreased, inhibiting stop codon recognition by eRF1 and promoting more incidents of readthrough [121, 123]. Ribosomal readthrough has been observed in response to oxygen and glucose deprivation, resulting in the translation of target protein isoforms [124]. While the functions of these protein isoforms translated in hypoxia are largely unknown, subunits or domains that contribute to hypoxic protein regulation and activation may exist in the additional protein sequence that confer differential regulation in hypoxia.

### HIF inhibitors as potential therapeutics

Modulating HIF activity is an area of interest in many different diseases including anemia, ischemia and cancer. In treating anemia and ischemia, increased HIF activity is favorable and patients are administered PHD inhibitors or HIF stabilizers, such as vadadustat, to increase HIF expression [125, 126]. Vadadustat is an investigational drug in Phase III trials to treat anemia. It that works by increasing HIF activity and consequently increasing erythropoietin and red blood cell production [127]. While HIFs play an integral role in cell survival under hypoxic stress, their dysregulation may result in cancer development and progression. In healthy cells, HIF1α expression is generally higher than HIF2α expression, except for in the pancreas, liver and kidneys. However, this relatively conserved HIF1α-to-HIF2α expression ratio is significantly higher or lower in many malignant solid tumors that express either more HIF1α or HIF2α than in normoxia. This imbalance is indicative of poor prognosis in patients [25, 26]. Targeting HIFs in cancers has been a growing area of interest that has entered the realm of clinical trials in the past decade, with some therapies showing potential, but none having yet received regulatory approval.

One major HIF1α-targeting small molecule inhibitor, PX-478, has demonstrated potent antitumorigenic effects [128]. It was found to significantly decrease HIF1α mRNA and protein levels by blocking its transcription and translation [128]. Furthermore, PX-478 treated cells have decreased rates of HIF1α de-ubiquitination, resulting in higher HIF1α degradation [128]. HIF1α target gene expression also decreased. In another study, PX-478 re-sensitized prostate carcinoma cells to radiation therapy [129]. PX-478 has undergone Phase I clinical trials for the treatment of advanced solid tumors or lymphomas and considering the positive results, the drug remains of interest for further evaluation as a cancer therapeutic [130].

Hypoxia-activated prodrugs are currently in development for clinical use. The benefit of hypoxia-activated prodrugs is the selectivity in targeting hypoxic cancer cells. One such prodrug is Evofosfamide (TH-302) has been proven to be especially effective in targeting cancer cells. Evofosfamide is a 2-nitroimidazole prodrug that undergoes a single electron reduction, resulting in a radical anion prodrug that immediately reacts with oxygen present in the environment [131]. This reaction reverts the anion prodrug back to the original state of Evofosfamide. In hypoxia, there is no oxygen molecules to interact with the anion prodrug, resulting in the fragmentation of the anion prodrug that results in the active alkylating cytotoxic agent [131]. This agent crosslinks DNA thus inhibiting replication. Due to the highly selective and potent effect of this drug on hypoxic cells, it has been used in Phase II clinical trials in combination with Bortezomib, a standard chemotherapeutic, in targeting hypoxic cancers in patients with relapsed myeloma [132]. The results show that the treatment combination was well tolerated in patients with modest efficacy [132].

While prodrugs may seem promising, the use of small molecules to target hypoxic cancer cells nevertheless appears to be effective. For example, the use of topotecan and other topoisomerase I inhibitors have been used to treat cancers in the clinic. The effect that topotecan has on hypoxia has been studied in clinical trials [133]. In 2011, the inhibitory effects of topotecan on HIF1α activity were evaluated in 22 patients [133]. Topotecan inhibits HIF1α by a mechanism independent of its role in DNA replication [133–135]. However, results showed no exclusive correlation between HIF target gene expression and topotecan treatment in patient cancer cells. While HIF expression and activity decreased in patients treated with topotecan, the expression of the HIF target gene *VEGF* was unchanged [133]. The results of this study did not suggest topotecan as a HIF-targeting cancer therapeutic due to its short plasma half-life of ~ 3 h, lack of HIF specificity and high toxicity [133, 136].

In 2016, the idea of using a nanoparticle conjugate CRLX101 with the administration of an anti-angiogenesis antibody bevacizumab to target hypoxic cancer cells was explored in a Phase I clinical trial [137]. The CRLX101 nanoparticle-drug conjugate is infused with a topotecan analog camptothecin, another topoisomerase I inhibitor. Camptothecin decreases HIF protein transcription, effectively decreasing its activity in hypoxic cells [137]. The reasons for using nanoparticle-drug conjugate to deliver camptothecin is two-fold. First, nanoparticles appear to preferentially aggregate into tumor cells, allowing for increased specificity in targeting cancer cells [137]. Second, the nanoparticle conjugate allows for a slow release of the infused camptothecin, significantly increasing the half-life of the drug [137]. Camptothecin also displays less toxicity compared to topotecan and is better tolerated by patients. The nanoparticle-drug conjugate CRLX101 is currently in several preclinical studies and Phase I and Phase II clinical trials for the treatment of gastroesophageal cancer, advanced renal cell carcinoma and breast cancer [138–141]. The effect that camptothecin has on hypoxic protein synthesis has not yet been studied.

There are also compounds that specifically target HIF2α activity, such as PT2385 and PT2399. PT2385 and PT2399 are both small-molecule antagonists that block the dimerization of HIF2α with ARNT by directly binding to the PAS domain of HIF2α, inhibiting the transcription of HIF2α target genes [142, 143]. The role of these small molecule inhibitors on HIF2α-mediated translation remain unreported. When tumor xenografts were treated with PT2385 in mice, HIF2α target gene expression significantly decreased in vitro and in vivo and HIF2α mRNA and protein expression levels also decreased in vivo. As a result, PT2385 treated tumor xenografts showed tumor regression, reduced angiogenesis, lower rates of cell proliferation and increased apoptosis. Based on the promising in vitro and in vivo studies, PT2385 was the first HIF2α antagonist to enter clinical trials and is currently in Phase II. While HIF2α transcriptional activity and expression levels is inhibited by PT2385, the effect of the drug on HIF2α translational role in hypoxia remains to be studied.

Another method of targeting hypoxic cancer cells is by inhibiting eIF4E2 activity. eIF4E2 is active only in hypoxia and complexes with HIF2α/RBM4 to initiate the first step of hypoxic translation [112]. By inhibiting eIF4E2, and consequently inhibiting hypoxic protein synthesis, cancer cells can be distinctively targeted from healthy cells by inhibiting the hypoxic protein synthesis pathway. Evidence suggests that eIF4E2 suppression significantly slows or even reverses cancer growth [112]. While an eIF4E2 targeting drug has immense potential as a cancer therapy, there has been difficulty finding a compound that can distinctively target eIF4E2 over eIF4E. There are currently therapies targeting eIF4E, such as the use of antisense oligonucleotides and small molecule inhibitors that block eIF4E complexing with eIF4G [144–146]. However, because these targeting methods cannot effectively distinguish eIF4E2 from eIF4E, healthy cells that utilize the cap-dependent translation initiation will also become the target of these therapies. Therefore, there is still a need to identify a cancer therapy that specifically targets eIF4E2 to inhibit protein synthesis in hypoxic cancer cells.

Targeting HIFs specifically in cancer cells may present an insurmountable challenge. Although a major hallmark in cancers, HIFs also have important roles in normal physiology and function of different tissues, such as normal kidney and liver which utilize hypoxia and the activation of HIF pathways to maintain homeostasis. Targeting HIFs, therefore, may inevitably lead to intolerably severe side effects. Furthermore, many HIF inhibitors target both HIF1α and HIF2α or are mechanistically aimed at inhibiting HIF transcriptional activity [22]. Developing a HIF2α-mediated translation specific inhibitor holds some potential to differentiate from currently available inhibitors. However, the lack of useful compounds targeting HIF2α-mediated translation makes it difficult to answers these questions.

## Summary

Cell stress initiated by a hypoxic environment necessitates intricate orchestration and reorganization of cellular homeostasis in order to adapt and survive such a harsh insult. While it is well known that the transcriptional landscape of the cell is changed, it is becoming clearer that hypoxic protein synthesis is also fine-tuned by oxygen-dependent proteins, such as HIFs and PHDs. Targeting hypoxic translational activity holds significant potential for the treatment of cancer, perhaps even more than targeting transcriptional activity due to the unique machinery cells use in protein synthesis for hypoxia adaptation.

## Abbreviations

4E-BP: Eukaryotic initiation factor 4E binding protein
AMPK: 5′ adnosine monophosphate activated protein kinase
ARNT: Aryl hydrocarbon receptor nuclear translocator
ATP: Adenosine triphosphate
bHLH: Basic helix-loop-helix
CBP: CREB-binding protein
Cdk1: Cyclin dependent kinase 1
Cdk2: Cyclin dependent kinase 2
C-MYC: Cancer myelocytomatosis gene
C-TAD: C-terminal transactivation domain
CXCR4: CXC chemokine receptor type 4
eEFs: Eukaryotic elongation factors
eIFs: Eukaryotic initiation factors
EPO: Erythropoietin
eRFs: Eukaryotic release factors
FDA: Food and Drug Administration
FGF: Fibroblast growth factor
FIH: Factor inhibiting Hypoxia-inducible factor
GADD34: Protein phosphatase 1 regulatory subunit 15A; also known as PPP1R15A
GDP: Guanine diphosphate
GLUT1: Glucose transporter 1
GTP: Guanine triphosphate
GTPase: Guanine triphosphatase
HIF: Hypoxia-inducible factor
HIFalpha: Hypoxia-inducible factor subunit alpha
HIFbeta: Hypoxia-inducible factor subunit beta
HIF-PH2: Hypoxia-inducible factor prolyl hydroxylase 2
HRE: Hypoxia response element
IGF: Insulin-like growth factor
IRES: Internal ribosome entry site
Jmjd4: Jumonji domain-containing 4
LC3C: Microtubule-associated proteins 1A/1B light chain 3C; also known as MAP1LC3C
LDH: Lactate dehydrogenase
mAUG: Main AUG
met-tRNAi: Methionine charged transfer ribonucleic acid
mORF: Main open reading frame
mRNA: Messenger ribonucleic acid
mTOR: Mammalian target of rapamycin
NIKS: Asparagine-Isoleucine-Lysine-Serine
N-TAD: N-terminal transactivation domain
OCT4: Octamer-binding transcription factor 4
ODDD: Oxygen dependent degradation domain
p300: E1A-associated protein 300
P4HA1: Proyly 4-hyroxylase subunit Alpha 1
PABP: Poly(A)-binding protein
PAS: Per-Arnt-Sim; Period circadian protein-aryl hydrocarbon receptor nuclear translocator protein-single-minded protein
PDGF: Platelet-derived growth factor
PERK: Protein kinase R (PKR)-like endoplasmic reticulum kinase
PHD: Prolyl hydroxylase domain
PIC: Pre-initiation complex
pO2: Oxygen gas partial pressure
pVHL-E3: von-Hippel Lindau tumor suppressor, E3 ubiquitin ligase complex
REDD1: DNA damage inducible transcript 4; also known as DDIT4
rHRE: Ribonucleic acid hypoxia response element
RMB4: RNA binding motif protein 4
SQRDL: Sulfide quinone reductase-like
SRP: Signal recognition particle
tRNA: Transfer ribonucleic acid
uAUG: Upstream AUG
uORF: Upstream open reading frame
UTR: Untranslated region
VEGF: Vascular endothelial growth factor
